# Berberine alleviates hyperglycemia by targeting hepatic glucokinase in diabetic *db/db* mice

**DOI:** 10.1038/s41598-019-44576-7

**Published:** 2019-05-29

**Authors:** Meng Li, Yanqi Dang, Qiong Li, Wenjun Zhou, Jianping Zuo, Zemin Yao, Li Zhang, Guang Ji

**Affiliations:** 1 Institute of Digestive Diseases, China-Canada Center of Research for Digestive Diseases (ccCRDD), Longhua Hospital, Shanghai University of Traditional Chinese Medicine, 200032 Shanghai, China; 2 Laboratory of Immunology and Virology, Shanghai University of Traditional Chinese Medicine, 201203 Shanghai, China; 3 Department of Biochemistry, Microbiology & Immunology, Ottawa Institute of Systems Biology, University of Ottawa, K1H 8M5 Ottawa, Canada

**Keywords:** Type 2 diabetes, Drug development

## Abstract

Berberine (BBR) is a widely used anti-diabetic agent, and liver glucokinase (GK) has been reported to be involved. However, the mechanisms of BBR in regulating GK are still unknown. Here, we found that BBR upregulated GK immunofluorescence expression in AML12 cells cultured in high glucose and increased glycogen content simultaneously. BBR improved hyperglycemia in *db/db* mice, and increased liver glucose-6-phosphate/glucose-1-phosphate (G-6-P/G-1-P) was found by analyzing metabolites (serum, liver, and feces) based on gas chromatography-mass spectrometry (GC-MS) metabolomics. Pharmacokinetics-pharmacodynamics (PK-PD) assessment revealed enriched BBR distribution in the liver, and liver G-6-P had the same trend as the concentration-time curve of BBR. G-6-P is solely catalyzed by GK, and GK activity and expression showed a positive correlation with liver BBR levels. In *db/db* mice, BBR also upregulated GK in liver fractions (cytoplasm and nucleus) and liver glycogen content. GK functionally worked by dissociating from GK regulatory protein (GKRP), and although GKRP expression was not affected, we found a decreased ratio of GK binding with GKRP in BBR treated *db/db* mice. In conclusion, our study suggests the dissociation of GK from GKRP as the potential mechanism for liver GK increase upon BBR treatment, which contributes to the anti-diabetic effect of BBR.

## Introduction

The emergence of type 2 diabetes mellitus (T2DM) as a global pandemic is one of the major challenges to human health in the 21st century^1^. In 2015, 415 million people suffered from diabetes worldwide (IDF, 2015)^2^, and diabetes is estimated to be a heavy burden on health care in future decades.

The liver plays a crucial role in controlling glucose homeostasis by coordinating the metabolism, synthesis, storage, and redistribution of nutrients^3,4^. In postprandial status, the liver contributes to the disposal of enteral glucose loads by enhancing glycogen synthesis and suppressing hepatic glucose export^5^. In the fasting state, the liver produces glucose by glycogenolysis to maintain euglycemia. These physiological processes are dysregulated in T2DM, and this imbalance contributes to hyperglycemia in both postprandial and fasting states^6^. Hepatic glycogen thus delicately coordinates the release of glucose from the liver and regulates the homeostasis of glucose metabolism.

The transport of glucose into hepatocytes occurs through glucose transporter type 2 (GLUT2). Glucose can freely exit hepatocytes without transformation, and once it enters the cell, glucose will transform into glucose-6-phosphate (G-6-P) to limit its outflow. The transformation of glucose to G-6-P in hepatocytes is catalyzed solely by glucokinase (GK), which works as the gatekeeper for glucose metabolism in hepatocytes. G-6-P can be either further transformed to glucose-1-phosphate (G-1-P) and synthesized into glycogen for storage or utilized by the cell via the tricarboxylic acid cycle and pentose phosphate pathway. The activator of GK is reported to increase liver glucose uptake in male Sprague-Dawley and Zucker diabetic fatty rats^7^, indicating that GK could be a promising target for hyperglycemia. GK activity is largely regulated by its subcellular localization. Physiologically, GK regulatory protein (GKRP) binds to GK in the nucleus when concentrations of glucose are low, and dissociation occurs under high-glucose conditions; however, this coordination is damaged in diabetic status^8,9^.

Control of T2DM is urgent, and available agents include insulin sensitizers^10^, insulinotropic peptides^11^, and glucose absorption inhibitors^12^, among others. However, these strategies have limitations, and the long-term effect is far from ideal, making new agents greatly needed. In recent years, the interest in natural products that treat T2DM has grown. Berberine (BBR, C_20_H_18_NO_4_), a pharmacological component principally isolated from *Rhizoma coptidis* Franch. (family *Ranunculaceae*), which has long been widely used in traditional Chinese medicine for treating infectious diarrhea, has now been identified as a potential treatment for T2DM. Both clinical investigations and animal studies have confirmed its role in lowering blood glucose and improving related metabolic disorders^13,14^.

While the effect of BBR on T2DM has been confirmed, numerous studies have reported the possible mechanisms of BBR according to respective design and experiment data, making the mechanisms underlying the efficacy of BBR debatable. In alloxan-induced diabetic mice, it was reported that BBR increased GK expression and activity^15^, indicating GK as the possible target of BBR. However, this finding has not been verified by other studies, and the regulation mechanisms are unknown. In the present study, we used AML12 cells and diabetic *db/db* mice to investigate GK expression upon BBR treatment, applied metabolomics, pharmacokinetics-pharmacodynamics (PK-PD) assessment and molecular biological techniques to explore the regulatory mechanisms of BBR on GK. Our data might provide a systematic understanding of GK regulation under the anti-diabetic effect of BBR.

## Results

### BBR increased GK expression and glycogen content in AML12 cells

**Figure 1:**
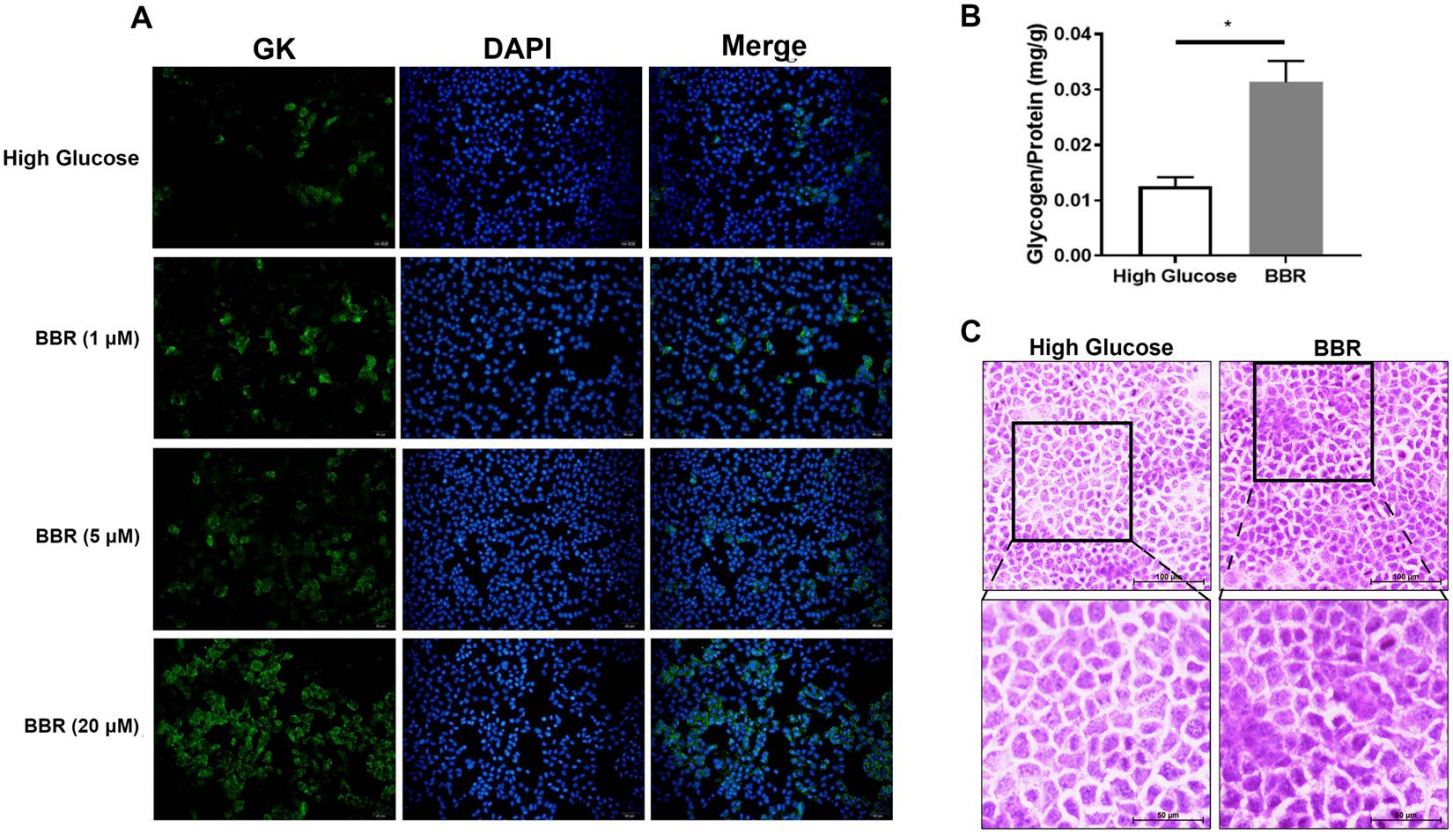
BBR promoted GK expression in AML12 cells. AML12 cells were maintained in high-glucose medium (17.5 mM) for 24 h in the presence or absence of BBR (1 μM, 5 μM, or 20 μM). Immunofluorescence of GK expression in cells was visualized (**A**). AML12 cells were treated with 20 μM BBR for 24 h, and glycogen content was chemically evaluated (**B**) and stained with PAS (**C**). Data are presented as the mean ± SEM, and the experiments were performed in triplicate. *P < 0.05 between groups.

To investigate the role of BBR in GK expression, we cultured AML12 cells in high-glucose medium, treated the cells with BBR (1 μM, 5 μM, or 20 μM) for 24 h, and observed the GK fluorescence intensity. This revealed that 20 μM BBR significantly increased GK expression, while the effect of low concentration (1 μM and 5 μM) BBR on GK was negligible (Fig. 1A). GK is the catalyzing enzyme of glucose metabolism in hepatocytes and contributes to glycogen synthesis. We also detected glycogen content, and glycogen content was significantly increased with 20 μM BBR treatment (Fig. 1B). Periodic acid Schiff (PAS) staining of the cells further confirmed increased glycogen distribution under microscope observation (Fig. 1C).

### BBR alleviated hyperglycemia in *db/db* mice

**Figure 2:**
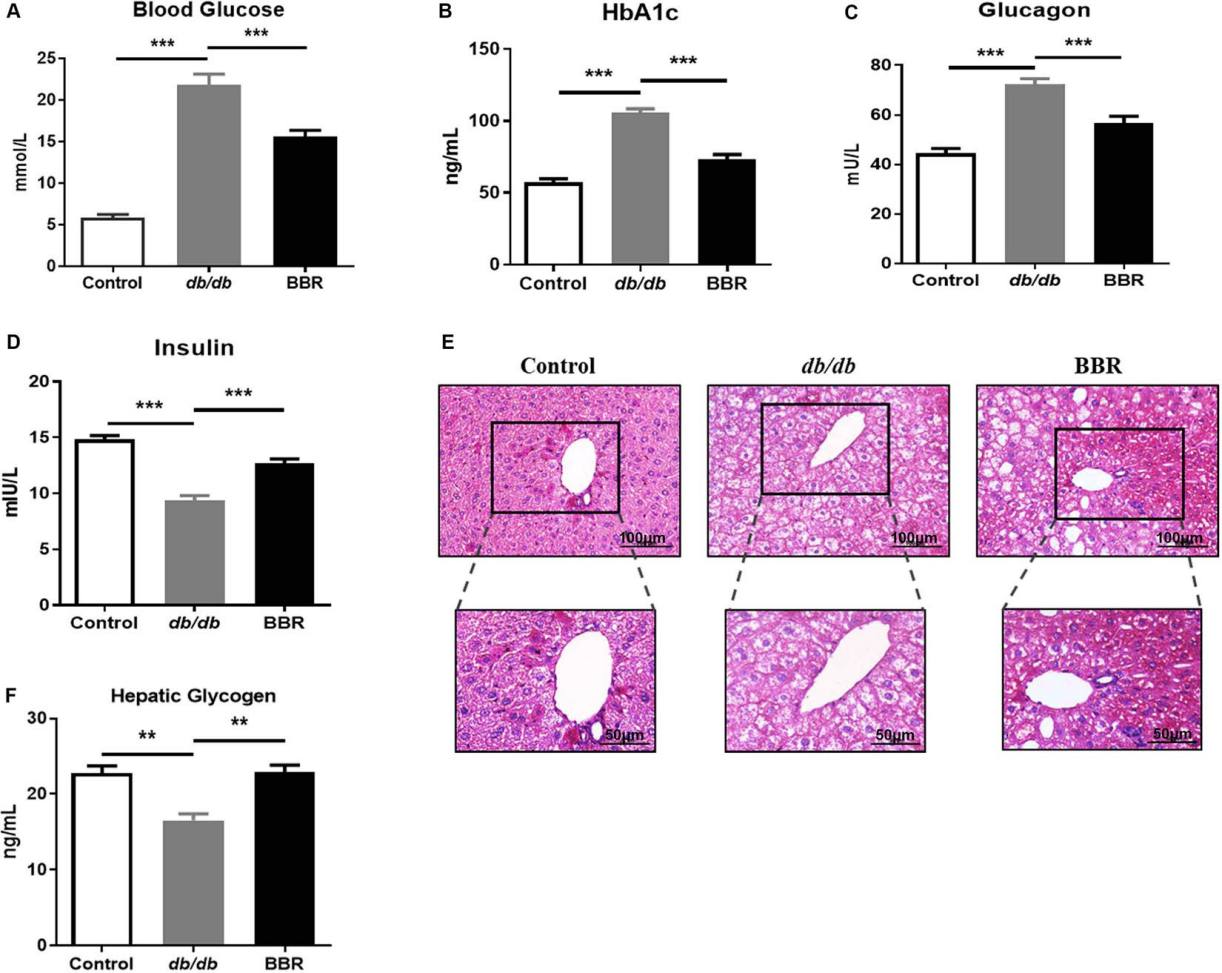
BBR alleviated hyperglycemia in *db/db* mice. Eight-week-old male *db/db* mice were treated with BBR (210 mg·kg^−1^·day^−1^) for 4 weeks, with untreated *db/db* mice and wild-type C57BL/6J mice used as controls. Blood glucose (**A**), HbA1c (**B**), glucagon (**C**) and insulin (**D**) levels were determined. Liver glycogen content (**E**) and representative PAS staining images (**F**, magnification was 200x and magnification of representative areas was 400x) were shown. Data are presented as the mean ± SEM, n = 8 per group. **P < 0.01 and ***P < 0.001 between groups.

To evaluate the effect of BBR on diabetic *db/db* mice, we administered BBR to *db/db* mice for four weeks. The untreated *db/db* mice exhibited a diabetic phenotype, with average fasting blood glucose, hemoglobin A1c (HbA1c) and glucagon levels showing 2.7-fold, 1.5-fold, and 0.84-fold increases in comparison with the wild-type control mice (*P* < 0.001). In contrast, BBR treatment significantly reduced these parameters, suggesting the anti-diabetic property of BBR (*P* < 0.001, Fig. 2A–C). Unexpectedly, we observed a remarkable decrease in serum insulin levels in *db/db* mice, and BBR significantly restored this decrease (Fig. 2D).

Liver glycogen serves as a form of energy storage and plays a pivotal role in regulating blood glucose. According to the PAS staining, glycogen deposition in the liver of *db/db* mice was significantly reduced, and 4-week BBR treatment significantly increased liver glycogen content (Fig. 2E). Biochemical analysis also confirmed the effect of BBR on liver glycogen increase (Fig. 2F).

### BBR altered the glucose-related metabolites in *db/db* mice

To explore the possible mechanisms underlying the efficacy of BBR, we conducted metabolomics in serum, feces and liver samples of the mice. Differential metabolites were found between *db/db* mice and wild-type mice; glucose-related metabolites in serum (e.g., galactonic acid, arabitol, ribitol, xylitol, maltose, glycerol and sedoheptulose), feces (lactic acid, glucose, ribose, fructose, rhamnose, arabinose and lyxose) and the liver (fructose-6-phosphate, dihydroxyacetone phosphate, glycerate-3-phosphate, glucose, ribose-5-phosphate, gluconic acid, arabitol, galactonic acid, fructose, sedoheptulose and galacturonic acid) were significantly increased in *db/db* mice (Supplementary Table S1).

**Figure 3:**
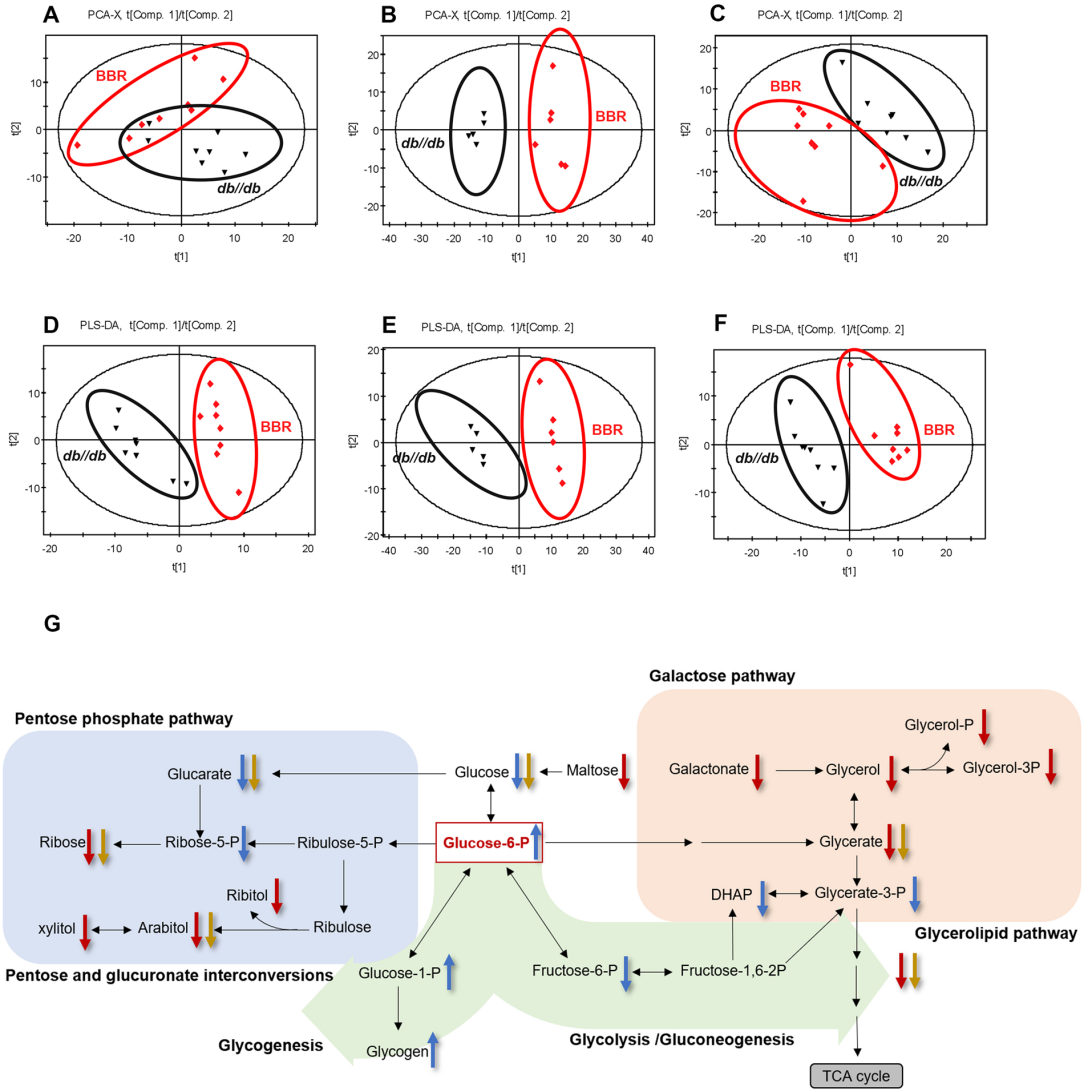
Metabolomics analysis of serum, liver and fecal samples. Metabolomics analysis was performed by GC-MS; PCA score plot of serum (**A**), liver (**B**) and feces (**C**), and PLS-DA score plot of serum (**D**), liver (**E**) and feces (**F**) from BBR-treated and untreated *db/db* mice are shown. (**G**) Glucose-related metabolites are summarized. Arrows pointing up and down represent increased and decreased metabolites, respectively, upon BBR treatment. Red arrows indicate changes in serum, blue indicates liver, and yellow indicates feces. Data are presented as the mean ± SEM, n = 8 per group. DHAP, dihydroxyacetone phosphate; Glucose-6-P, Glucose-6-phosphate; Glycerate-3-P, Glycerate-3-phosphate; Glycerol-2-P, Glycerol-2-phosphate; Glycerol-3-P, Glycerol-3-phosphate; Fructose-6-P, Fructose-6-phosphate; Fructose-1,6-2P, Fructose-1,6-diphosphate; Ribose-5-P, Ribose-5-phosphate; Ribulose-5-P, Ribulose-5-phosphate.

We next compared the glucose-related metabolites between BBR-treated and untreated mice, and a score plot of principal component analysis (PCA) completely separated the metabolites (serum *R*^2^*X* = 0.717, *Q*^2^ = 0.283, feces *R*^2^*X* = 0.524, *Q*^2^ = 0.166 and liver *R*^2^*X* = 0.527, *Q*^2^ = 0.0945) of the two groups (Fig. 3A–C). Distinctly separated clusters were also shown by the partial least squares-discriminant analysis (PLS-DA) model (Fig. 3D–F). The evaluation parameters *R*^2^*X*, *R*^2^*Y* and *Q*^2^ were 0.434, 0.923 and 0.687 in serum; 0.489, 0.995 and 0.933 in feces; and 0.378, 0.962 and 0.819 in liver samples, respectively. BBR-treated mice had different metabolic profiles from untreated mice. 12 metabolites (sucrose, maltose, ribitol, arabitol, glycerol-3-phosphate, xylitol, glycerol-2-phosphate, ribose, galactonic acid, pyruvic acid, glyceric acid, and glycerol) in serum, 6 in feces (glucose, gluconic acid, ribose, arabitol, glyceric acid and pyruvic acid) and 7 in liver samples (glucose, G-6-P/G-1-P, fructose-6-phosphate, gluconic acid, ribose-5-phosphate, dihydroxyacetone phosphate, and glycerate-3-phosphate) were significantly different between BBR-treated and untreated groups (Fig. 3G). BBR treatment decreased most of the glucose-related metabolites, which was consistent with the hypoglycemic effect (Table 1). However, hepatic G-6-P/G-1-P were found to be increased upon BBR intervention.

**Table 1:** Glucose-related metabolites of serum, liver and feces from BBR-treated and untreated *db/db* mice.

Metabolites	Formula	Mol Weight	p-value	q-value	FC
**Serum**					
Maltose	C_12_H_22_O_11_	342.30	9.22E-03	1.81E-02	−1.12
Ribitol	C_5_H_12_O_5_	152.15	1.73E-02	2.56E-02	−0.98
Xylitol	C_5_H_12_O_5_	152.15	5.78E-03	1.58E-02	−0.61
Ribose	C_5_H_10_O_5_	150.13	1.48E-02	2.37E-02	−0.53
Arabitol	C_5_H_12_O_5_	152.15	7.24E-03	1.70E-02	−0.81
Galactonic acid	C_6_H_12_O_7_	196.16	1.48E-02	2.37E-02	−0.55
Glyceric acid	C_3_H_6_O_4_	106.08	2.81E-02	3.41E-02	−0.37
Pyruvic acid	C_3_H_4_O_3_	88.06	2.19E-02	2.85E-02	−0.49
Glycerol-3-phosphate	C_3_H_9_O_6_P	172.07	3.11E-03	2.85E-02	−0.66
Glycerol-2-phosphate	C_3_H_9_O_6_P	172.07	5.50E-04	3.1E-03	−0.57
Glycerol	C_3_H_8_O_3_	92.09	3.17E-02	3.43E-02	−0.35
**Liver**					
Glucose	C_6_H_12_O_6_	180.16	1.58E-03	8.4E-03	−0.27
Glucose-6-phosphate	C_6_H_13_O_9_P	260.14	7.45E-03	1.99E-02	0.90
Fructose-6-phosphate	C_6_H_13_O_9_P	260.14	1.59E-02	2.83E-02	−0.37
Gluconic acid	C_6_H_12_O_7_	196.16	3.51E-02	4.45E-02	−0.28
Ribose-5-phosphate	C_5_H_11_O_8_P	230.11	1.49E-02	2.75E-02	−0.4
DHAP	C_3_H_5_O_6_P	168.04	2.85E-03	1.05E-02	−0.95
Glycerate-3-phosphate	C_3_H_6_O_7_P	185.05	2.07E-02	3.31E-02	−0.23
**Feces**					
Glucose	C_6_H_12_O_6_	180.16	4.33E-03	9.80E-03	−1.55
Gluconic acid	C_6_H_12_O_7_	196.16	4.07E-02	4.18E-02	−2.55
Ribose	C_5_H_10_O_5_	150.13	4.33E-03	9.80E-03	−4.11
Arabitol	C_5_H_12_O_5_	152.15	7.60E-04	6.00E-03	−0.75
Glyceric acid	C_3_H_6_O_4_	106.08	8.66E-03	1.52E-02	−1.51
Pyruvic acid	C_3_H_4_O_3_	88.06	4.33E-03	9.80E-03	−6.96

Note: ^a^Comparison of differential glucose related metabolites between BBR-treated and untreated *db/db* mice with a Student’s *t* test, and all p-values were after FDR correction; FC: fold change was calculated as a binary logarithm of the average mass response (normalized peak area) ratio between BBR-treated and untreated *db/db* mice, where a positive value means that the average mass response of the metabolite in BBR-treated *db/db* mice is larger than that in untreated *db/db* mice.

### PK-PD assessment

**Figure 4:**
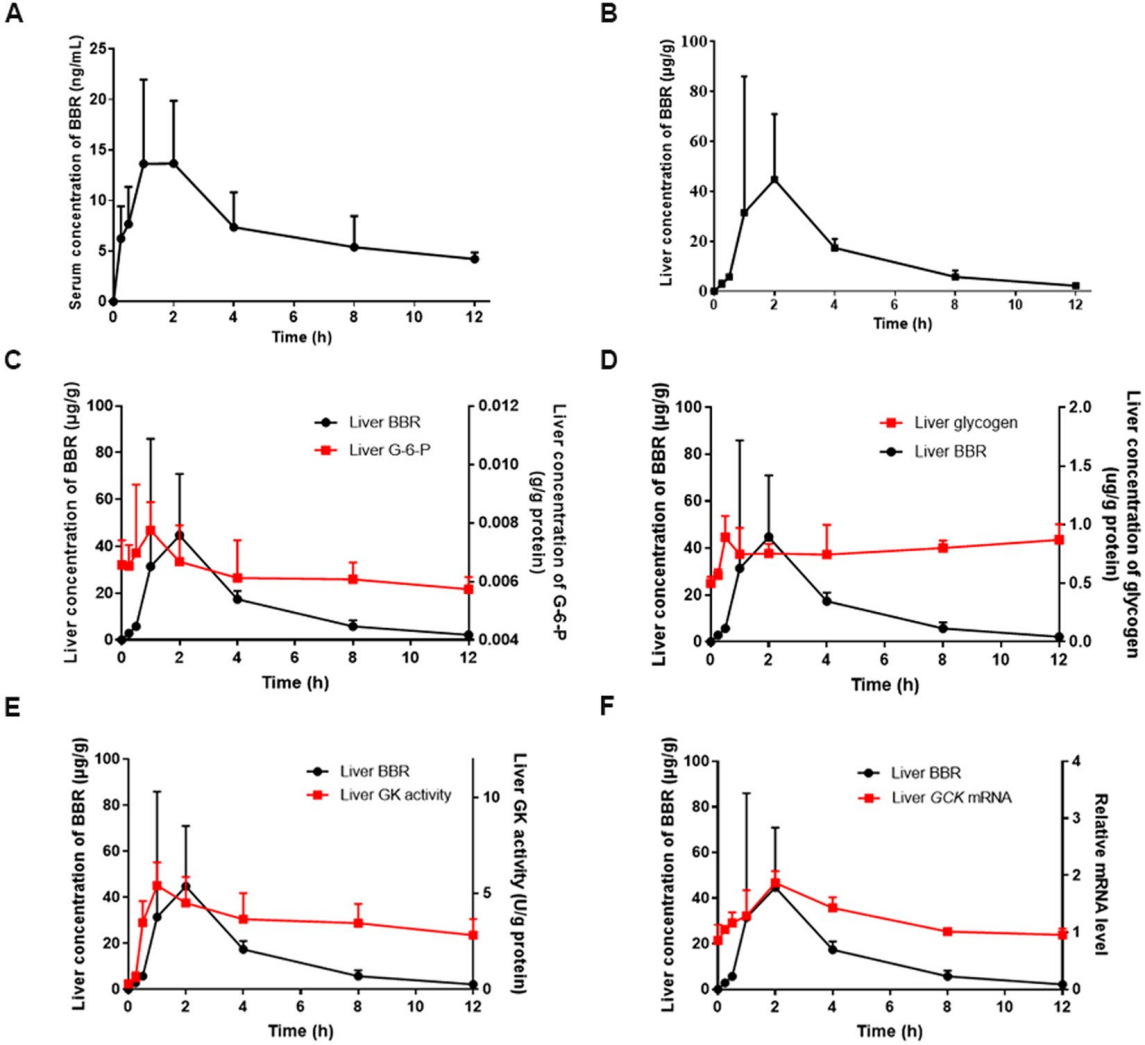
PK-PD assessment. Eight-week-old male *db/db* mice were randomly divided into 8 groups (n = 5 per group) and sacrificed at 0.25, 0.5, 1, 2, 4, 8, and 12 h after BBR gavage (210 mg·kg^−1^·day^−1^). The untreated mice (0 h) were used as controls. Concentration-time curves of serum (**A**) and liver (**B**) BBR are shown. Liver G-6-P (**C**) and glycogen (**D**) concentration-time curves, GK activity-time curve (**E**), and *GCK* expression-time curve (**F**) were drawn along with the concentration-time curve of liver BBR. The data are presented as the mean ± SEM, n = 5 per group.

An *In vivo* pharmacokinetic study in *db/db* mice treated with BBR was performed. The mean serum and liver concentration-time curves are shown in Fig. 4A,B. The maximum concentration (*C*_*max*_) of serum BBR was 13.66 ng·mL^−1^, the time to reach *C*_*max*_ (*T*_*max*_) was 2 h and the area under the concentration-time curve (AUC_0-t_) was 87.26 ng·h·mL^−1^ (Supplementary Table S3). In contrast, liver *C*_*max*_ was 44.73 μg/g, *T*_*max*_ was 2 h and AUC_0-t_ was 173.00 μg·h·g^−1^ (Supplementary Table S4), indicating that orally administered BBR rapidly reached the liver and had higher concentrations.

We compared the liver concentrations of G-6-P and glycogen with liver BBR at each sampling time point. G-6-P reached the maximal concentration 1 h earlier than the *T*_*max*_ of liver BBR (Fig. 4C), and glycogen content increased with the administration of BBR (Fig. 4D). G-6-P is catalyzed by GK, as the distribution of GK is mainly in the liver, and the association of GK and liver BBR levels was also analyzed. The curves of GK activity (Fig. 4E) and mRNA expression (Fig. 4F) showed the same trend as the liver concentration-time curve of BBR and had a positive correlation (correlation factor was 0.473 and 0.873, respectively). Notably, the maximal enzyme activity of GK was 1 h, which was earlier than the *T*_*max*_ of liver BBR. These data suggested that GK activity and expression were sensitive to changes in liver BBR levels.

### BBR upregulated hepatic GK expression in diabetic *db/db* mice

**Figure 5:**
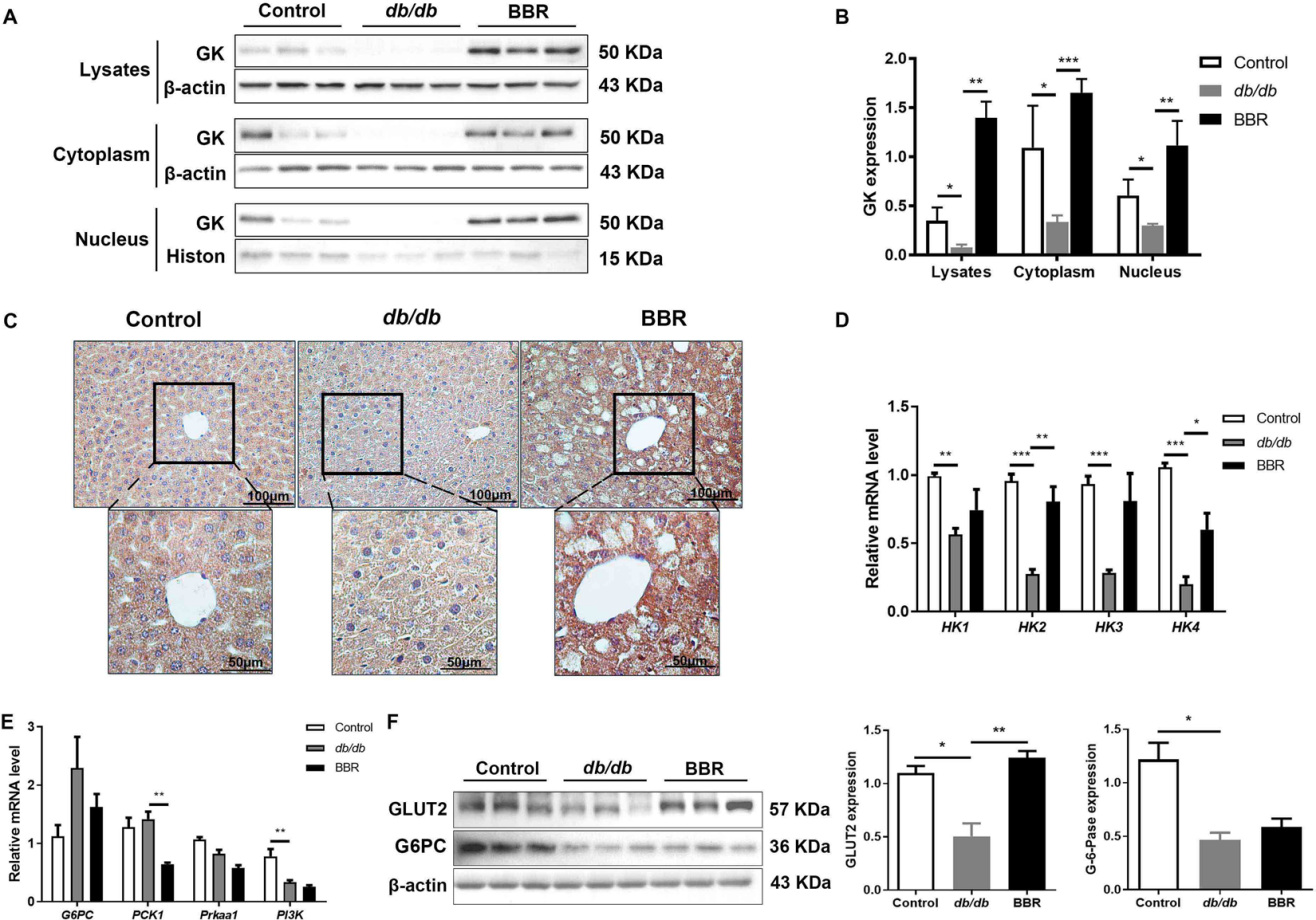
BBR upregulated hepatic GK expression in *db/db* mice. Western blot analysis (**A**) and qualification (**B**) of GK in liver lysates, cytoplasm and nucleus. (**C**) Immunohistochemistry staining for GK in liver sections. The mRNA levels of *HK1-4*, *G6PC*, *PCK1*, *Prkaa1* and *PI3K* were quantified by qRT-PCR (**D**,**E**). (**F**) Hepatic GLUT2 and G-6-Pase were detected by Western blot. The groupings were cropped from different gels subjected to identical conditions. Data are presented as the mean ± SEM, n = 3 per group. ^*^P < 0.05, ^**^P < 0.01, and ^***^P < 0.001 between groups.

GK is distributed both in the nucleus and cytoplasm, but only cytoplasmic GK functions, so we detected GK protein in liver lysates, cytoplasm and nucleus. The expression of GK in *db/db* mice was significantly decreased, and BBR treatment restored its expression in all liver portions (Fig. 5A,B). To further observe GK expression, we applied immunohistochemistry staining with GK antibody, and GK-positive areas showed a significant increase in BBR-treated mice compared to the untreated *db/db* mice (Fig. 5C).

GK is encoded by *GCK* (also known as *hexokinase IV*, *HK4*), and we analyzed the mRNA expression of the four *HKs* (*HK1-4*) in the liver. *HK1-4* expression was remarkably decreased in *db/db* mice, and BBR treatment restored *HK4* mRNA expression, which was consistent with the change in GK expression. *HK2* mRNA expression also increased upon BBR treatment (Fig. 5D). In addition, we analyzed gluconeogenesis-related glucose-6-phosphatase (*G6PC*), phosphoenolpyruvate carboxykinase (*PCK1*), AMPKla (*Prkaal*) and phosphoinositide 3-kinase (*PI3K*), and only *PCK1* expression was suppressed upon BBR treatment (Fig. 5E). We also observed a significant decrease in GLUT2 in *db/db* mice, and BBR partially restored GLUT2 expression (Fig. 5F).

### BBR facilitated GK-GKRP dissociation

**Figure 6:**
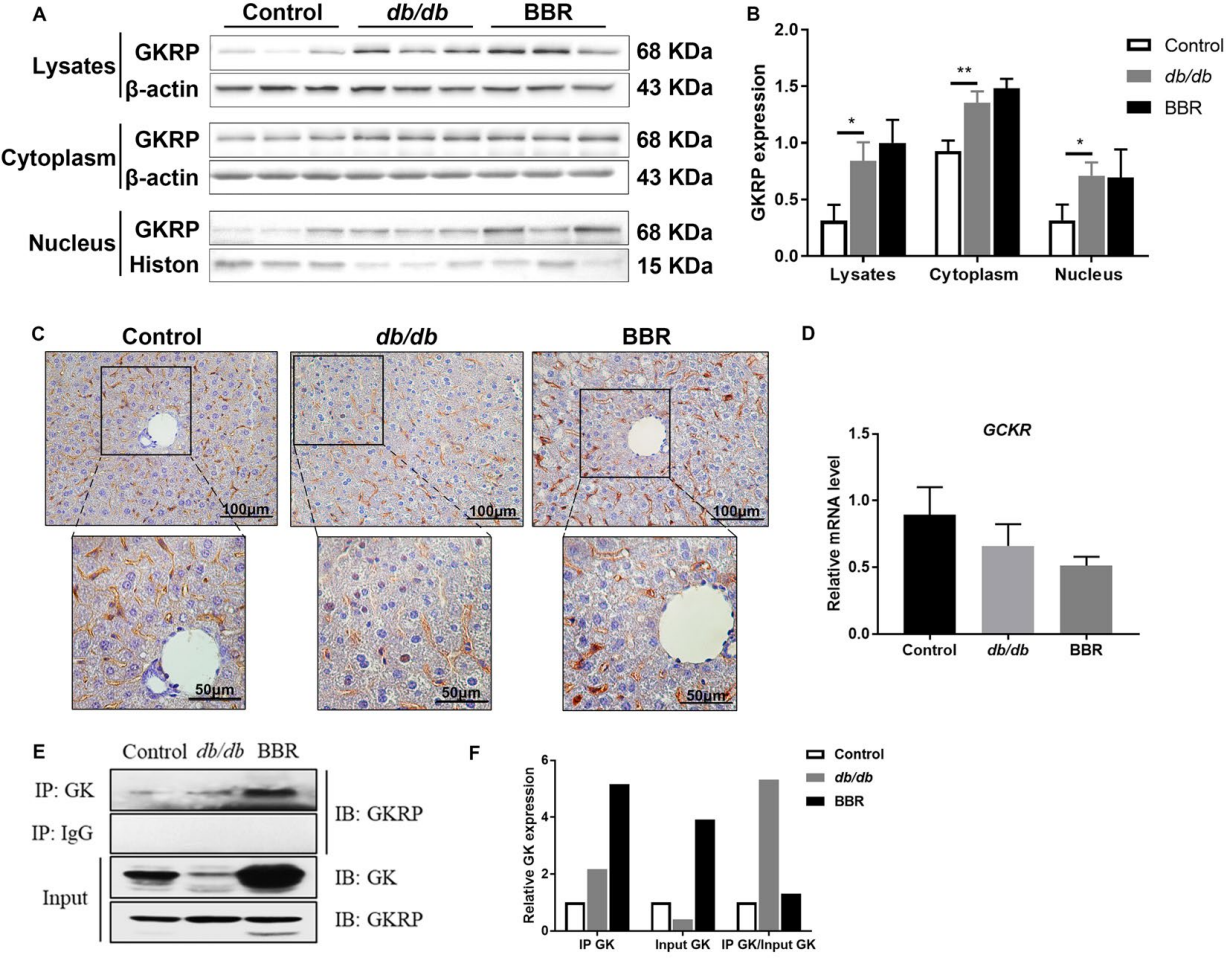
BBR increased GK-GKRP dissociation. Western blot analysis (**A**) and qualification (**B**) of GKRP in liver lysates, cytoplasm and nucleus. (**C**) Immunohistochemistry staining for GKRP in liver sections. (**D**) The mRNA level of *GCKR* was quantified by qRT-PCR. Liver lysates were immunoprecipitated (**E**) and quantified (**F**) by an anti-GK antibody, and immunoblotting was performed to detect the binding status of GK and GKRP. The groupings were cropped from different gels subjected to identical conditions. Data are presented as the mean ± SEM, n = 3 per group. ^*^P < 0.05 and ^**^P < 0.01 between groups.

Studies have demonstrated that the activity of GK is regulated by direct binding with GKRP. We also observed increased GKRP in the liver of *db/db* mice; however, the effect of BBR on liver GKRP at the transcriptional and translational levels was negligible (Fig. 6A–D). To further evaluate the binding status of GK and GKRP, we applied a Co-IP experiment, and with identical protein input, we observed that GK expression (Input GK) decreased, GK binding with GKRP (IP GK) increased, and the ratio of IP GK (GK-GKRP binding form)/Input GK increased in *db/db* mice compared with wild-type mice, whereas BBR treatment decreased the ratio, suggesting more GK released from GKRP and could function in glucose metabolism (Fig. 6E,F).

## Discussion

**Figure 7:**
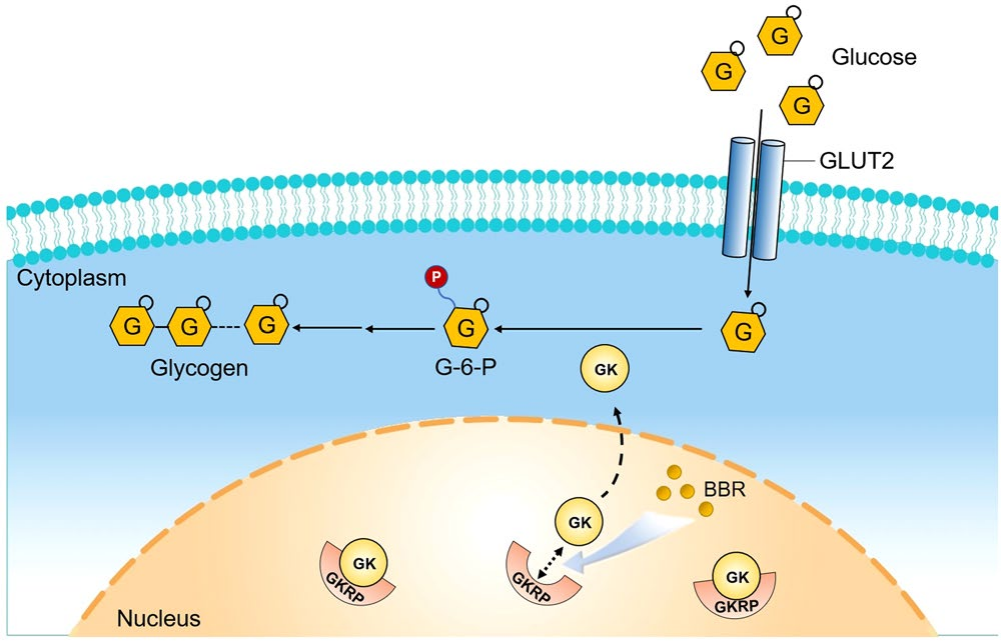
Potential mechanisms of GK increase due to the anti-diabetic property of BBR. Through increasing GK release from GKRP, BBR upregulated hepatic GK expression and increased glucose uptake and glycogen content in the liver, thus exerting an anti-diabetic effect in *db/db* mice.

In the present study, we confirmed that BBR upregulates GK expression in high-glucose cultured AML12 cells and showed the hypoglycemic effect of BBR in *db/db* mice. We obtained increased liver G-6-P/G-1-P through metabolomics detection. PK-PD assessment revealed that both GK activity and expression were positively correlated with liver BBR levels. The increase in liver GK and glycogen content was further verified in BBR-treated *db/db* mice. We also showed increased dissociation of GK from GKRP with BBR treatment. Our results indicated that increased GK release was one of the potential mechanisms of BBR in treating diabetes (Fig. 7).

The glucose-lowering effect of BBR has long been studied. In the 1980s, Chinese researchers accidentally discovered that T2DM patients took BBR as a folk recipe to control their blood glucose, and a small sample investigation further confirmed its effect^16^. From that time, studies of BBR as a T2DM remedy have spread globally. In 2008, a pilot study identified that 3-month BBR treatment could significantly decrease blood glucose and HbA1c in newly diagnosed T2DM patients^17^. Yan *et al*. further confirmed that the anti-diabetic effect of BBR is superior to that of placebo in T2DM patients^18^. The anti-diabetic effects of BBR have also been confirmed in various animal models with T2DM characteristics^19–21^.

Although the therapeutic efficacy of BBR in T2DM was confirmed, the underlying mechanisms of its efficacy are debated due to different study designs, animal strain or race, and patterns of modeling. Diabetic *db/db* mice are the most commonly mice used in T2DM studies. Researchers have also found that adenosine 5′-monophosphate-activated protein kinase (AMPK) in adipocytes and muscle can be activated by BBR, thus promoting peripheral glucose uptake and contributing to its hypoglycemic effect^22^. BBR has also been reported to activate AMPK in macrophages and other peripheral tissues to alleviate metabolic-related stress in *db/db* mice^23,24^. By orally administering BBR to *db/db* mice, Li *et al*. found that BBR improved diabetic characteristics by increasing SIRT1 protein expression and alleviating endoplasmic reticulum stress^25^. In high-fat diet fed ICR mice, BBR is reported to inhibit SIRT3 to reduce hepatic glucose production^26^. Hepatic gluconeogenesis is also supported by BBR in diabetic status, and key gluconeogenesis enzymes, such as PCK and G6PC, can be reduced by BBR, indicating the inhibition of gluconeogenesis upon BBR intervention^14,27^.

Metabolomics have been developed and widely applied in biological studies since the 1990s. In studying the mechanisms of BBR in diabetes, metabolites of samples from both T2DM patients and animals have been analyzed, and certain fatty acids were identified to be involved^18,28^. Here we noticed the alteration in G-6-P/G-1-P and identified GK as the target by retrieval and verification. Hepatic GK expression is physiologically regulated by fasting and refeeding cycles and is pathologically reduced in diabetes^29,30^. The levels of insulin, glucagon and glucocorticoids all affect the activity of GK, while GK activation works dramatically on glucose metabolic pathways^31–33^. The accumulated G-6-P/G-1-P due to GK activation could be further transformed into glycogen or utilized for energy. Corresponding to the upregulation of G-6-P/G-1-P, we also observed increased hepatic glycogen content upon BBR treatment in *db/db* mice, which restored insufficient glycogen reserve in diabetic status, consistent with previous studies^15,34^. Excessive glycogen in the liver is correlated with numerous diseases, such as glycogen storage disease type I (GSD I), also known as Von Gierke disease, for which deficiency of specific enzymes is the main cause^35^. Although some gastrointestinal symptoms occurred in BBR clinical trials^36,37^, no reports of BBR-related liver glycogen excess were found.

Previous studies showed that GKRP posttranscriptionally regulates GK function in hepatocytes via binding and dissociating processes^38,39^. The GKRP seemed unaffected by BBR at both the transcriptional and translational levels. Such an inconsistency might be due to the animals or feeding, and the sample processing methods may also be related. Another study also reported that the deacetylation of GKRP contributes to the facilitation of hepatic glucose uptake^8,38,40^. Our data suggest that GK is the dominant target of BBR in regulating hepatic glucose uptake.

In conclusion, we confirmed an increase in liver GK and hypoglycemia upon BBR treatment. GK activity and expression were positively related to liver BBR levels, and GK increase was associated with GK release from GKRP, which contributed to the anti-diabetic effect of BBR.

## Methods

### Materials

BBR hydrochloride (purity ≥99%) was purchased from Shanghai Ronghe Pharmaceutical Technology Development Company (Shanghai, China). L-leucine-^13^C_6_-^15^N, L-isoleucine-^13^C_6_-^15^N, L-valine-^13^C_5_-^15^N, L-alanine-^13^C_3_-^15^N, methoxylamine hydrochloride, dulcitol and N,O-bis(trimethylsilyl)-trifluoroacetamide (BSTFA) with 1% trimethylchlorosilane (TMCS) were purchased from Sigma-Aldrich (St. Louis, MO, USA). Methanol, ethanol and isopropanol for HPLC grade were purchased from Merck Chemicals (Darmstadt, Germany). Chloroform of analytical grade was purchased from Sinopharm Chemical Reagent Company (Shanghai, China). Trizol reagent was purchased from Invitrogen (Carlsbad, CA, USA).

### Animals

Eight-week-old male C57BL/6 J wild-type (20-25 g, n = 10) and leptin receptor-mutated (C57BL/KsJ-*db/db)* mice (35–40 g, n = 18) were purchased from the Model Animal Research Center of Nanjing University (Nanjing, China). Animals were acclimatized in specific pathogen-free rooms at a temperature of 20–26 °C, humidity of 30–70% and a 12-h light/dark cycle for one week. All animal experiments were performed in accordance with the approved guidelines of the Experimental Animal Care and Ethics of Animal Experiments Committee of Shanghai University of Traditional Chinese Medicine (Shanghai, China).

After one week of acclimatization, *db/db* mice were randomly divided into two groups based on blood glucose: the untreated group (*db/db* group, n = 8) and the BBR treatment group (BBR group, n = 10). BBR (210 mg·kg^−1^·day^−1^) was suspended in 0.5% sodium carboxy methylcellulose (CMC-Na) solution and then administered to the treated mice, while the untreated mice were given an equal volume of 5% CMC-Na solution for 4 weeks. Regular laboratory chow and filtered tap water were allowed ad libitum, and the mice fasted 12 h before sacrifice.

### Biochemical analysis of serum and liver tissue

After four weeks of treatment, the mice were anesthetized with 2% sodium pentobarbital, blood was obtained and the serum was separated by centrifugation for 15 min at 3000 rpm. Levels of insulin, glucagon and HbA1c were measured using mouse ELISA kits (Shanghai Enzyme-linked Biotechnology Company, Shanghai, China). Blood glucose was measured by an automated biochemical analyzer.

The liver was rapidly removed, weighed, and washed with precooled normal saline, and a fraction of the same position was cut and fixed in 10% formalin, while remaining fractions were stored at −80 °C after being snap-frozen in liquid nitrogen. Liver glycogen was analyzed using an assay kit (Dongou Technologies Company, Zhejiang, China) according to the manufacturer’s instructions and read with UV-mini1240 spectrophotometer (Shimadzu, Kyoto, Japan).

### Liver histological and immunohistochemical analysis

Liver fractions were fixed in formalin overnight and embedded in paraffin wax. Paraffin sections (4 μm) were prepared for PAS (Solarbio, Beijing, China) staining to observe the pathological changes and glycogen storage. For immunohistochemical analysis, liver sections were pretreated with citrate buffer, incubated with anti-GK (Santa Cruz Biotechnology, sc-17819) and anti-GKRP (Santa Cruz Biotechnology, sc-166841) antibodies overnight at 4 °C, and then polymerized horseradish peroxidase-conjugated secondary antibodies were added to visualize the positive area. Images were captured using a system incorporated in a Nikon Eclipse 50i microscope with magnification of 200x, and representative areas were further shown with magnification of 400x.

### Metabolomic profile

The process of sample preparation, gas chromatography-mass spectrometry (GC-MS) analysis, data processing, bioinformatics and statistical analysis were conducted as previously described^41^. In brief, the serum, liver and feces were prepared, added with internal standards, dried under a gentle nitrogen stream, incubated with methoxylamine hydrochloride in anhydrous pyridine, derivatized with BSTFA (with 1% TMCS) and then splitlessly injected into an Agilent 7890 A series GC coupled to an HP-5MS column (30 m × 0.25 mm, 0.25 film thickness) and an Agilent 5975 C inert MSD detector. The GC-MS data were processed with DataBridge (Perkin-Elmer, USA), and multivariate statistical analysis was applied with SIMCA-P 11.0 software (Umetrics AB, Umeå, Sweden) to perform PCA and PLS-DA. The differential metabolites and metabolic pathways were determined using the Golm Metabolome and KEGG Database.

### Immunoblotting

Liver tissues were homogenized in ice-cold radioimmune precipitation (RIPA) lysis buffer (Beyotime Institute of Biotechnology, Shanghai, China) with protease and phosphatase inhibitors. Nuclear and cytoplasmic fractions were obtained using the nuclear and cytoplasmic protein extraction kit (Beyotime). Protein lysates were separated on 10% SDS-PAGE, transferred to polyvinylidene difluoride (PVDF) membranes (Millipore, Temecula, CA), and immunoblotted with the following primary antibodies: GK (sc-17819, 1:1000), GKRP (sc-166841, 1:1000) and G-6-Pase (sc-15840, 1:1000), which were purchased from Santa Cruz Biotechnology (CA, USA), and an antibody against GLUT2 (ab544460, 1:1000), which was purchased from Abcam (Cambridge, MA, USA). As a loading control, β-actin (4970, 1:1000) was purchased from Cell Signaling Technology (Beverly, MA, USA) and histone (ab8580, 1:1000) was purchased from Abcam. For secondary antibodies: anti-mouse IgG (14709, 1:3000) and anti-rabbit IgG (5127, 1:3000) were obtained from Cell Signaling Technology. Blot bands were quantified using the Chemi Fluorescent and Chemiluminescent Imaging System (Syngene, Cambridge, UK). The groupings of the blots cropped from different gels subjected to identical conditions, and the full-length gels are shown in Supplementary Fig. S1.

### Co-Immunoprecipitation (Co-IP)

Liver tissue was lysed with RIPA buffer, centrifuged, and precleared with protein A/G agarose beads (Santa Cruz). Supernatants were collected and immunoprecipitated overnight with anti-GK antibody (sc-17819, Santa Cruz) or negative control IgG antibody (Boster Biol Tech, Wuhan, China). After overnight incubation, protein A/G agarose beads were added, pelleted by centrifugation, washed three times with ice-cold washing buffer, boiled for 5 min, and subjected to electrophoresis, and GKRP expression was analyzed by immunoblotting. The groupings of the blots cropped from different gels subjected to identical conditions, and the full-length gels are shown in Supplementary Fig. S1.

### Quantitative RT-PCR

Total RNA was extracted from liver tissue using Trizol reagent. cDNA was reverse-transcribed with the high capacity reverse transcription kit from Applied Biosystems (Monza, Italy) and used in RT-PCR with SYBR Green PCR Master Mix (Toyobo, Japan) in a StepOne Applied PCR system. The data were analyzed by the 2^−ΔΔCt^ method, and samples were normalized to glyceraldehyde-3-phosphate dehydrogenase (GAPDH). The primer sequences are listed in Supplementary Table S2.

### Cell culture and glycogen storage detection

Mouse AML12 hepatocytes were obtained from the Cell Bank of the Chinese Academy of Sciences (Shanghai, China), and cultured in Dulbecco’s modified Eagle’s medium (DMEM)/F12 (GIBCO, 11330-032) supplemented with 10% (v/v) fetal bovine serum (FBS), 1% (v/v) ITS liquid media supplement (sigma, I3146) and 40 ng/ml dexamethasone (Sigma, D4902). The cells were incubated at 37 °C in an incubator continuously supplying 5% CO_2_.

After incubation of mouse AML12 hepatocytes without or with 20 μM BBR for 24 h at 37 °C, we determined glycogen content with hepatocyte ELISA kits (Shanghai Enzyme-linked Biotechnology Company). Protein content was measured as well, and glycogen content was expressed as mg/g protein. Cells were fixed with 4% formaldehyde and a PAS staining kit (Solarbio) was used to evaluate glycogen storage.

### Immunofluorescence

AML12 hepatocytes were treated with or without BBR (1, 5, or 20 μM). The cells were then fixed with 4% paraformaldehyde (w/v), permeabilized with 0.5% Triton X-100, and incubated with blocking buffer containing 10% bovine serum albumin (BSA) for 2 h at room temperature. Next, the cells were incubated overnight at 4 °C with GK antibody containing 10% BSA, washed with phosphate buffer saline (PBS), and incubated in the dark for 2 h with Alexa Fluor 488-conjugated goat anti-mouse secondary antibodies (Invitrogen). After washing with PBS, the nucleus was stained with 2-(4-amidinophenyl)-6-indolecarbamidine dihydrochloride (DAPI) for 10 min, and images were taken by an Olympus IX71 fluorescence microscope.

### PK-PD study of BBR in *db/db* mice

Eight-week-old male C57BL/KsJ-*db/db* mice were randomly divided into 8 groups (n = 5 per group) and sacrificed at 0.25, 0.5, 1, 2, 4, 8, and 12 h after BBR gavage (210 mg·kg^−1^·day^−1^). The untreated mice (0 h) were used as controls. All animals were fasted 12 h before administration of BBR. At each time point, animals were anesthetized, and blood and liver tissues were collected.

10% liver tissue homogenate of *db/db* mice was prepared by adding 10 mL saline to 1 g liver tissue. A 50-μL aliquot of serum/homogenate and 200 μL acetonitrile containing 50 ng/mL IS was vortexed for 2 min and then centrifuged at 12,000 × g for 5 min. Then, 10 μL of the aliquot was injected into the liquid chromatography-tandem mass spectrometry (LC-MS/MS) system to analyze the concentration of BBR, and the method was performed as previously described^42^.

All calibration and quantitation data were processed with MassHunter Workstation Qualitative Analysis Software Version B.04.00. The serum/tissue concentration-time data were analyzed using noncompartmental methods with the software program PK solutions 2™ (Summit Research Services, USA) to determine pharmacokinetic parameters. A Pearson correlation analysis was performed using SPSS 18.0. Liver glycogen was analyzed, and G-6-P and GK activity were analyzed using an ELISA kit (Westang Biotechnology company, Shanghai, China). *GCK* expression was measured.

### Statistical analysis

Quantitative data are expressed as the mean ± SEM. Statistically significant differences were assessed using two-tailed Student’s *t*-test or one-way ANOVA. SPSS 18.0 statistical software (SPSS, Chicago, IL, USA) was used for statistical analysis. Values of *P* < 0.05 were considered statistically significant.

## Supplementary information

supplementary material

## Data Availability

All data generated or analyzed during this study are included in this published article (and its Supplementary Information file).
