# Supplementary material for: Berberine alleviates hyperglycemia by targeting hepatic glucokinase in diabetic db/db mice
Source: Sci Rep. 2019 May 29;9:8003. doi: 10.1038/s41598-019-44576-7 (PMC6541623; doi:10.1038/s41598-019-44576-7)
Supplement: Supplementary file 1 — supplementary material [file 41598_2019_44576_MOESM1_ESM.docx]

**Berberine alleviates** **hyperglycemia by targeting hepatic** **glucokinase in diabetic *db/db* mice**

Meng Li^1^, Yanqi Dang^1^, Qiong Li^1^, Wenjun Zhou^1^, Jianping Zuo^2^, Zemin Yao^3^, Li Zhang^1*^, Guang Ji^1*^

^1^Institute of Digestive Diseases, China-Canada Center of Research for Digestive Diseases (ccCRDD), Longhua Hospital, Shanghai University of Traditional Chinese Medicine, Shanghai 200032, China

^2^Laboratory of Immunology and Virology, Shanghai University of Traditional Chinese Medicine, Shanghai 201203, China

^3^Department of Biochemistry, Microbiology & Immunology, Ottawa Institute of Systems Biology, University of Ottawa, Ottawa K1H 8M5, Canada

*Correspondence author

Li Zhang (zhangli.hl@163.com) & Guang Ji (jiliver@vip.sina.com)

Tel.: +86-021-64385700

**Supplementary Table 1.** **Glucose-related metabolites of serum, liver and feces from wild-type and *db/db* mice**

| **Serum** | | | | | |
| --- | --- | --- | --- | --- | --- |
| **Metabolites** | **Formula** | **Mol Weight** | **p-value** | **q-value** | **FC** |
| Gluconic acid | C_6_H_12_O_7_ | 196.16 | 9.00E-05 | 2.40E-04 | 1.31 |
| Galactonic acid | C_6_H_12_O_7_ | 196.16 | 1.00E-05 | 3.56E-05 | -1.38 |
| Arabitol | C_5_H_12_O_5_ | 152.15 | 2.00E-04 | 4.74E-04 | -2.34 |
| Ribitol | C_5_H_12_O_5_ | 152.15 | 2.11E-03 | 3.11E-03 | -2.00 |
| Xylitol | C_5_H_12_O_5_ | 152.15 | 1.10E-03 | 1.82E-03 | -1.02 |
| Maltose | C_12_H_22_O_11_ | 342.30 | 4.80E-04 | 1.82E-03 | -3.89 |
| Glycerol | C_3_H_8_O_3_ | 92.09 | 1.00E-05 | 3.56E-05 | -0.66 |
| Sedoheptulose | C_7_H_14_O_7_ | 210.18 | 5.00E-05 | 1.45E-04 | -1.23 |
| **Liver** | | | | | |
| **Metabolites** | **Formula** | **Mol Weight** | **p-value** | **q-value** | **FC** |
| Fructose-6-phosphate | C_6_H_13_O_9_P | 260.14 | 2.00E-05 | 8.38E-05 | -1.00 |
| DHAP | C_3_H_5_O_6_P | 168.04 | 7.99E-03 | 8.85E-03 | -1.61 |
| Glycerate-3-phosphate | C_3_H_6_O_7_P | 185.05 | 7.99E-03 | 8.85E-03 | -0.35 |
| Glucose | C_6_H_12_O_6_ | 180.16 | 1.00E-05 | 4.79E-05 | -0.77 |
| Ribose-5-phosphate | C_5_H_11_O_8_P | 230.11 | 4.66E-03 | 5.58E-03 | -1.14 |
| Gluconic acid | C_6_H_12_O_7_ | 196.16 | 3.40E-04 | 9.90E-04 | -1.49 |
| Arabitol | C_5_H_12_O_5_ | 152.15 | 4.57E-03 | 5.57E-03 | -0.95 |
| Galactonic acid | C_6_H_12_O_7_ | 196.16 | 6.83E-03 | 7.89E-03 | -0.47 |
| Fructose | C_6_H_12_O_6_ | 180.16 | 1.00E-04 | 3.72E-04 | -2.20 |
| Sedoheptulose | C_7_H_14_O_7_ | 210.18 | 0.00E+00 | 0.00E+00 | -1.85 |
| Galacturonic acid | C_34_H_32_N_4_O_8_ | 624.64 | 7.60E-04 | 1.27E-03 | -0.92 |
| **Feces** | | | | | |
| **Metabolites** | **Formula** | **Mol Weight** | **p-value** | **q-value** | **FC** |
| Lactic acid | C_3_H_4_FeO_3_ | 143.91 | 4.33E-03 | 9.61E-03 | -0.83 |
| Glucose | C_6_H_12_O_6_ | 180.16 | 2.60E-04 | 2.53E-03 | -1.09 |
| Ribose | C_5_H_10_O_5_ | 150.13 | 8.26E-03 | 1.40E-02 | -1.39 |
| Fructose | C_6_H_12_O_6_ | 180.16 | 1.00E-05 | 1.40E-02 | -1.93 |
| Rhamnose | C_6_H_12_O_5_ | 164.16 | 1.73E-02 | 2.23E-02 | -0.67 |
| Arabinose | C_5_H_10_O_5_ | 150.13 | 3.02E-02 | 3.46E-02 | -1.60 |
| Lyxose | C_5_H_10_O_5_ | 150.13 | 3.91E-03 | 3.46E-02 | -2.81 |

Note: ^a^Comparison of differential metabolites between wild-type and *db/db* mice with a Student’s *t* test, and all p-values were after FDR correction; FC: fold change was calculated as a binary logarithm of the average mass response (normalized peak area) ratio between wild type-mice vs *db/db* mice, where a positive value means that the average mass response of the metabolite in wild-type mice is larger than that in *db/db* mice.

**Supplementary Table S2. Oligonucleotide primer sequences used for RT-PCR**

| **Target mRNA** | **Sequence (5′-3′)** |
| --- | --- |
| *HK1* | GTCAGAACGTCAGCATGGAGTC |
|  | CTCGTCTATTTTGGATTGTCGG |
| *HK2* | GTGACGGCGGTGGCTTAC |
|  | CCATTTCCACCTTCATCCTTC |
| *HK3* | GATGGGACACTCTACAAGCTACATC |
|  | CGACAAGCAACAGCAGTAACC |
| *HK4* | GTGTAAAGATGTTGCCCACCTAC |
|  | CGTCTTCACGCTCCACTGTC |
| *GCKR* | ATGCCCACCTACCAGCGAC |
|  | CAGACATAAGGAATGCCATACGAC |
| *G6pc* | ATCAATCTCCTCTGGGTGGC |
|  | TGTTGCTGTAGTAGTCGGTGTCC |
| *Pck1* | GATGTCGGAAGAGGACTTTGAG |
|  | CATAGGGCGAGTCTGTCAGTTC |
| *Prkaa1* | AAGTCAAAGCCGACCCAATG |
|  | TTCCTTCGTACACGCAAATAATAG |
| *PI3K* | AAGCCATTGAGAAGAAAGGACTG |
|  | ATTTGGTAAGTCGGCGAGATAG |
| *GAPDH* | TGTTCCTACCCCCAATGTGTC |
|  | TGAAGTCGCAGGAGACAACC |

**Supplementary Table S3. Serum pharmacokinetic of berberine in *db/db* mice**

| **Serum pharmacokinetics parameters** | **Berberine** |
| --- | --- |
| C_max_ (ng/mL) | 13.66 |
| T_max_ (h) | 2.00 |
| AUC_(0-t)_ (ng h/mL) | 87.26 |
| AUC_(0-∞)_ (ng h/mL) | 155.99 |
| T_1/2_ (h) | 11.29 |

**Supplementary Table S4. Liver pharmacokinetic of berberine in *db/db* mice**

| **Liver pharmacokinetics parameters** | **Berberine** |
| --- | --- |
| C_max_ (μg/mL) | 44.73 |
| T_max_ (h) | 2.00 |
| AUC_(0-t)_ (μg h/g) | 173.00 |
| AUC_(0-∞)_ (μg h/g) | 181.91 |
| T_1/2_ (h) | 2.84 |

**Supplementary Figure S1. Full-length gels for the cropped groupings of blots**


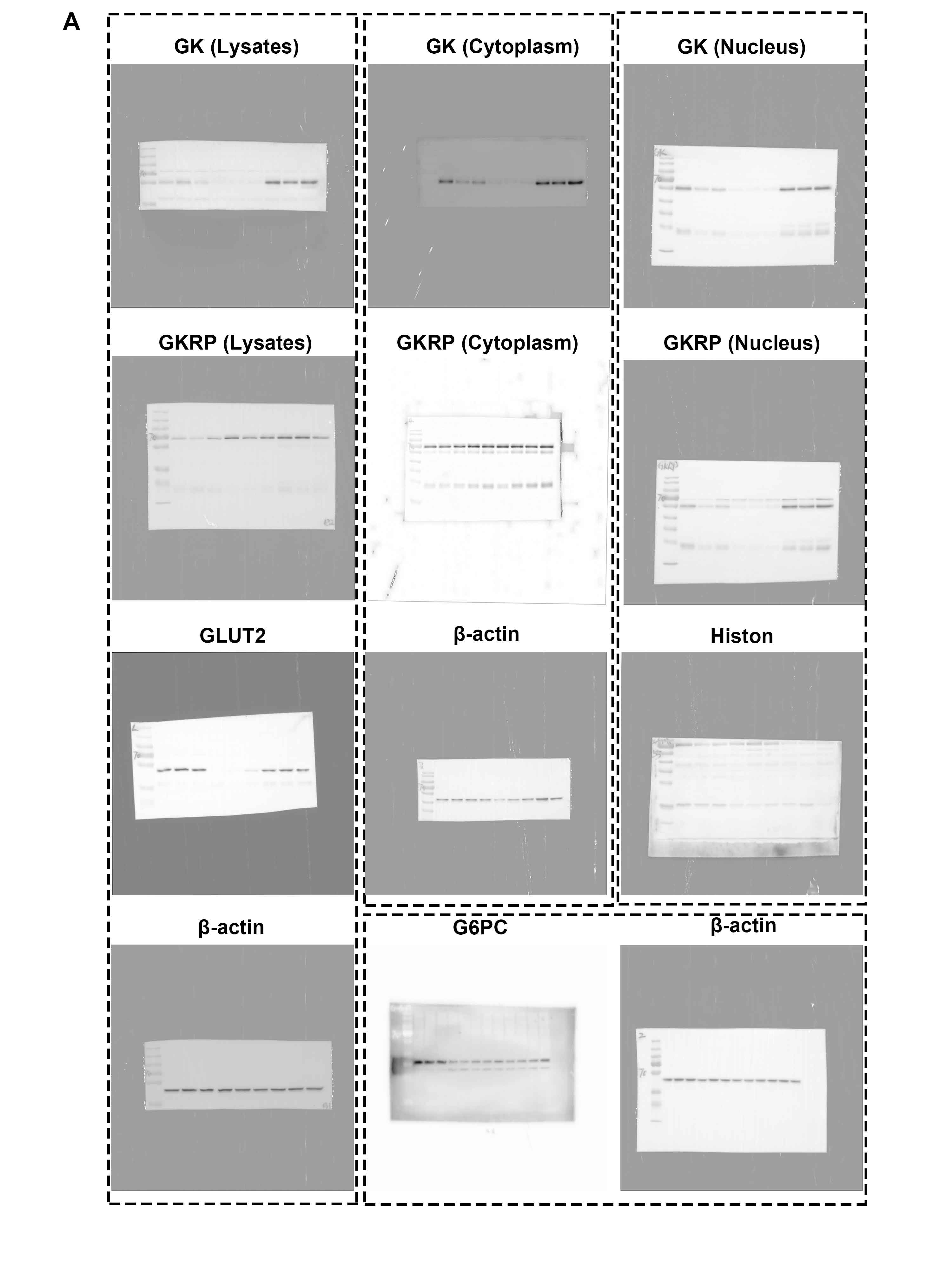


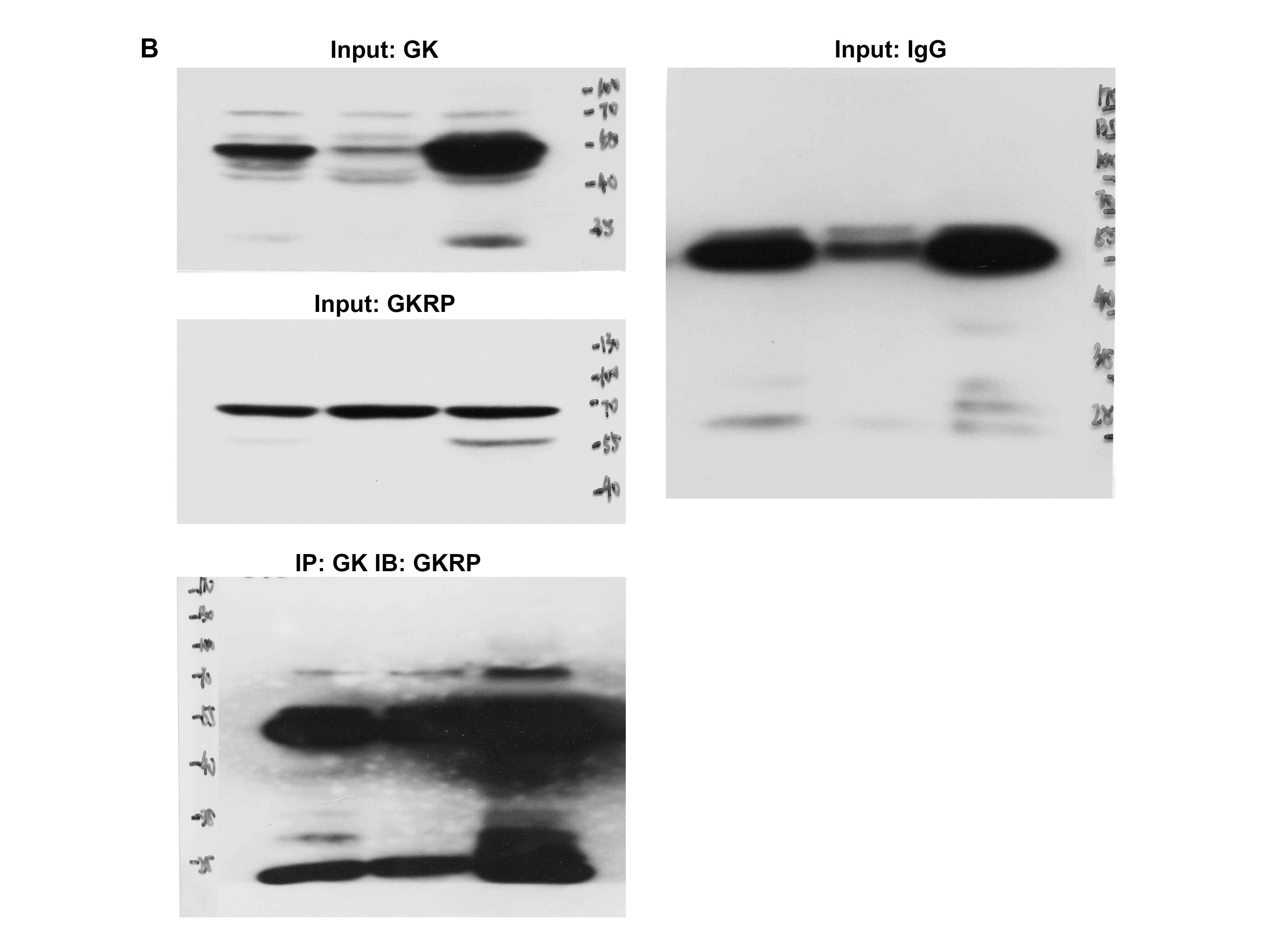


**Supplementary Figure S1. Full-length gels for the cropped groupings of blots. A** Full-length gels for the cropped groupings of blots in figure 3A, 3F and 4A; **B** Full-length gels for the cropped groupings of blots in figure 4E.
